# Supplementary material for: “When a story gets a face…”: Visual elicitation of Serbian perspectives on Afghan refugee women’s maternity experiences in Serbia
Source: PLOS Glob Public Health. 2024 Feb 15;4(2):e0002563. doi: 10.1371/journal.pgph.0002563 (PMC10868851; doi:10.1371/journal.pgph.0002563)
Supplement: Attachment — Submitted filename: Sharma-WEB-Responsetoreviewers_12102023.docx [file pgph.0002563.s001.docx]

Dear editors,

Thank you for considering our manuscript entitled “*“When a story gets a face…”:* visual elicitation of Serbian perspectives on Afghan refugee women’s maternity experiences in Serbia” [PGPH-D-23-00818] for publication in PLOS Global Public Health. We are grateful for the detailed comments from both reviewers that have helped us improve this manuscript. We have addressed comments point-by-point below, with relevant page numbers in brackets and highlighted changes in the revised manuscript in yellow.

Sincerely,

Study authors

**Detailed responses**

**Reviewer 1.0: Comments to Author - Thank you for the opportunity to review this article on a very important, global and topical issue. Please find a few comments below for your consideration. Could the authors please address the following;**

Response 1.0. Thank you for your kind words. We have worked to address your specific comments below.

**Reviewer 1.1. I was intrigued by the use of a likert scale with such a wide range of responses (10 in total). As you know, the use of likert scales has its challenges in terms of weighting/ and or the meaning of responses. It is unclear in the article how such an analyses was handled to arrive at the scoring and ranking of responses presented in Table 2. It seems mean scores were calculated but an explanation is not given on why means were the most appropriate measure to report. I am sure the authors know that a choice has to be made between reporting modes, medians and frequencies vs. means and standard deviations. The appropriate analyses is informed by the design considerations re: how the likert scale was structured.**

Response 1.1. Thank you for this comment. The ten-point scale we used was in fact a ranking scale, not a Likert scale, and we have corrected this in the text (p 9; line 212). We were interested in the mean average, to determine the typical ranking values. We have added a sentence to reflect the choice of mean score as: “ES analysed data from closed-ended questions descriptively, calculating frequencies and mean scores for ranking using Excel” (p 11; line 249).

**Reviewer 1.2. In the analysis, authors mention “cross tabulations”. It is unclear what cross tabulations were done, what test was this and the results thereof? It is unclear from the results of the quantitative data presented. Could authors classify- cross tabulations of what/which variables? See line 239-240**

Response 1.2. Thank you for raising this point. We don’t present any cross-tabulated data in the paper, so have removed this sentence.

**Reviewer 1.3. In line 273-272, the finding around mental health is an important aspect that needs to be highlighted in the discussion. The perinatal period is such a period in which many women develop depression even without the extended vulnerabilities of being a refugee, thus mental health of these women would have been hugely affected. Worth discussing how it is one of 2 categories that received the lowest ranking- perhaps something not very well understood by this population? Or it may speak to possible resilience and or coping mechanisms that, the lives lived by these women presents a constant and everyday worry that everything has become so internalised that they are not able to “amplify” the effects of mental health in a questionnaire? This goes back to my earlier comment about the likert scale and its ability to pick/” weight” particular issues.**

Response 1.3. We agree that it would be useful to discuss why support for mental health was ranked joint-lowest out of all aspects of maternal healthcare. We added a paragraph to address your point (p 21; lines 486-495)

**Reviewer 1.4. Re: qualitative findings, I had difficulties following some quotes and drawing meaning out of them as the authors sometimes put down a section of the quote and not a full one. for example, in line 359, it will be good to see more of the text/quote leading to "...saddened me the most"**

Response 1.4. Thank for this comment. We have added more of the text leading up to the quotes (p 16 lines 370 and 396-398).

**Reviewer 1.5. I found a few typos in the manuscript – e.g., see lines 455, 480, 526**

Response 1.5. Thank you for highlighting this. Several typos throughout the manuscript have now been corrected, including those that you pointed out.

**Reviewer 1.6. Re: limitations section, I find the fact that the survey was online (line 231-232) another form of selection bias- only those who had online access were able to consider/complete the survey. Authors should include this in their limitations section.**

Response 1.6. This is a useful point and we have added this into the Limitations section (p 23; lines 546-547).

**Reviewer 2.0. Comments to the Author: This is a very important study on the maternity experiences of a vulnerable population of Afghan women. The manuscript is well written and formatted. Although the study was generally well conducted, given that the researchers were working with vulnerable populations, there are a few important details that are missing from the manuscript. My specific comments are as follows:**

Response 2.0. Thank you for your helpful comments that we have tried to address below.

**Reviewer 2.1. Methods: You used a decolonial feminist approach to visualize participant lived experiences. Given that you interviewed Afghan women, does approach align with their mindset? Was this approach also used to analyse the data from the health professionals and community members?**

Response 2.1. Thank you for your very interesting comment. To address your last point first – we did not use a decolonial feminist lens to analyse the data from health professionals and CSOs. Moving to your first point, although we did not directly ask our participants what they thought of using a decolonial feminist lens during the webcomic development, we drew on other research exploring researching refugees to inform our choice of decolonial feminism as an approach (such as Pittaway et al 2010 <https://doi.org/10.1093/jhuman/huq004> and Singh et al <https://doi.org/10.1016/S0140-6736(21)00024-6>). Using a decolonial feminist lens was a challenge to *us* as researchers about how we conducted the research process, causing us to consider not only how we could centre and amplify Afghan women’s narratives, but also employing methodologies which would address power imbalances, such as narrative interviews (which were participant-led) and co-production (Lokot 2019 <https://doi.org/10.1080/13552074.2019.1664046>). We have expanded upon our application of the decolonial approach in the section “Theoretical stance for webcomic development” (lines 128-139).

**Reviewer 2.2. Lines 147-148: Please indicate the exact period when the webcomic was developed and tested.**

Response 2.2. Thanks. We have added these dates (p 7; lines 155-156).

**Reviewer 2.3. Lines 146-159: Please indicate the criteria that were used to select the 11 Afghan women that were interviewed e.g Afghan women who had recently delivered in Serbia. Please also briefly profile the women that were involved in the production of the comic. It is also not clear if the 5 participants that co-developed the comic were similar or different from the 6 other participants that only participated in the interview**

Response 2.3. We agree that adding details about the inclusion criteria would strengthen readers understanding of who participated in the narrative interviews and the webcomic development and have added this into the text (lines 153-354). We have stated that five of the eleven women who participated in narrative interviews joined in the co-production of the webcomic (lines 164-165). In terms of whether they are different or similar to those who participated in interviews but did not consent to join the co-production of the webcomic, we feel that any defining characteristics we may decide to assign to them, in order to ascertain similarities or differences would be arbitrary and would not add value to the understanding of how to webcomic was co-produced, and furthermore, we would feel uncomfortable categorising women for this purpose. We agree that it would be valuable to add a brief profile of the women who took part in the co-production of the webcomic, and have added this into the manuscript (lines 169-172).

**Reviewer 2.4. The study interviewed Serbian CSOs. Do Afghan civil society groups (formal or informal) exist in Serbia? If yes, then it would have also been good to include them in the study.**

Response 2.4. Thank you for this comment. To the best of our knowledge, there are no Afghan CSOs in Serbia, probably due to the highly transitory nature of the Afghan population there and we clarified this in the narrative (p 10, lines 237-8).

**Reviewer 2.5. Lines 219-228: In the introduction you indicated that registered Afghan refugees can access a range of healthcare services including maternity care. Unregistered refugees living in squatter camps can only access emergency care. Given the different types of healthcare services registered vs unregistered refugees can access, it would be good for you to describe the types of healthcare professionals that were interviewed in this study. Did these healthcare professionals work in maternity wards? Adding this information will contextualize the results**

Response 2.5. Thank you. We agree that this information would be valuable. However, with the knowledge that we had from field visits about the extreme reluctance and nervousness of health professionals to participate in research, and the great difficulties we had experienced in recruiting professionals to the wider research project, of which this is part, we were keen to limit the amount of personal information we collected from health professions, in order to maximise anonymity and increase our chances of recruiting as many health professionals as possible to complete this online questionnaire. We have acknowledged this in our Limitations section (p 23; lines 547-552).

**Reviewer 2.6. Line 242: In the methods section you indicated that you used reflexive thematic analysis and that ES developed the themes. Did the other authors also analyse the data and interpret the findings? Were the same researchers involved in the development of the web-comic and analysis of the data?**

Response 2.6. Thank you. We have clarified this as: “ES developed initial coding and themes and all authors contributed to interpreting findings” (p 10, lines 254-255).

**Reviewer 2.7. Discussion, Line 425-428: This study aimed to understand the awareness and attitudes of Serbian health professionals and members of women’s civil society organisations about the perinatal experiences of Afghan women in Serbia, using a webcomic to elicit responses. Therefore I suggest that you move line 434 to the first few sentences of the discussion (lines 425-428) as it highlights that most of the respondents were not aware of Afghan women's maternity experiences**

Response 2.7. Thank you for this suggestion – we have moved the sentence as you suggested (p 19; line 440).

**Reviewer 2.8. lines 513-521: There is potential selection bias because respondents needed access to an online survey.**

Response 2.8. Thank you – we have now addressed this in our Limitations section (lines 546-547).

**Reviewer 2.9. This study only asked questions on the healthcare experiences of Serbian women. Based on the results, it seems some of poor healthcare practices are experienced by all women in Serbia. Therefore the study could have benefited from asking general questions on maternity care in Serbia to contextualize the results**

Response 2.9. Thanks. We agree that exploring the maternity experiences of Serbian women is vital and warrants further research. However, at the time of developing the online questionnaire, it was outside the aims of our study, and we only became aware of the similarities between Afghan and Serbian women’s experiences retrospectively. We have thus added this to limitations (p 23; lines 552-555).

**Reviewer 2.10. Limited information is provided on the study participant characteristics. Additional information on who was included in the study (e.g. type of healthcare professional, maternal age, pregnant or postpartum woman etc.). would contextualize the results.**

Response 2.10. Thank you for this comment. We discussed participant characteristics of health professionals. With regards to the other suggestions of maternal age, pregnant or postpartum woman, there was no requirement in our inclusion criteria that participants should be women, be pregnant or have ever given birth.
